# Building Company Health Promotion Capacity: A Unique Collaboration Between Cargill and the Centers for Disease Control and Prevention

**Published:** 2009-03-15

**Authors:** Jason E. Lang, James C. Hersey, Christina M. Lynch, Karen L. Isenberg, Elizabeth Majestic

**Affiliations:** Office of the Director, National Center for Chronic Disease Prevention and Health Promotion, Coordinating Center for Health Promotion, Centers for Disease Control and Prevention; RTI International, Washington, DC; RTI International, Washington, DC; RTI International, Waltham, Massachusetts; Centers for Disease Control and Prevention, Atlanta, Georgia

## Abstract

**Background:**

The US Centers for Disease Control and Prevention (CDC) helps protect the health and safety of all people. The workplace can be used to reach millions of workers and their families with programs, policies, and benefits that promote health. We describe a CDC-led project to build Cargill's workplace health promotion capacity and identify the importance of a company liaison in the public-private relationship.

**Context:**

The project goals were to engage diverse Cargill personnel, conduct a workplace health assessment, aid in the development of a workplace health program action plan, and develop Cargill's internal capacity using knowledge and skill-building.

**Methods:**

CDC partnered with Cargill on a workplace health promotion project to build Cargill's capacity. A multicomponent assessment was conducted to determine priority employee health issues, stakeholder meetings were held to engage and educate Cargill management and employees, and technical assistance was provided regularly between CDC and Cargill.

**Consequences:**

Identifying a company liaison to work with an external assessment team is critical to building capacity for a successful workplace health project. This relationship creates an understanding of company culture and operations, facilitates access to key stakeholders and data, and provides opportunities to enhance capacity and sustainability.

**Interpretation:**

Employers undertaking workplace health promotion projects should identify a senior-level person to serve as the company health leader or liaison and who can devote the time necessary to build trusting relationships with partners to ensure project success. This person is valuable in facilitating communications, data collection, logistical support, troubleshooting, and influencing employer workplace health practices.

## Background

As the nation's premier public health agency, the Centers for Disease Control and Prevention (CDC) helps protect the health and safety of all people. The workplace can be used to reach millions of workers and their families with health promotion programs, policies, and benefits that promote health and prevent disease.

Increasing health care costs and decreased worker productivity are leading American businesses to examine strategies to improve employee health and contain health costs that are largely driven by chronic diseases and related lifestyle choices (1,2). Employers are recognizing the role they can play in creating a healthy work environment and providing their employees with opportunities to make healthy lifestyle choices. They increasingly look to CDC and other public health experts for guidance and solutions to combat the effects of  chronic diseases on their employees and businesses. Workplace health programs not only benefit individual employees but also make good business sense (3-5).

A top concern for many employers is health care costs. Employers are the nation's principal source of health insurance; they cover 62% of employees and their families and pay for 36% of all personal health care expenditures (6,7). Since 2001, health insurance costs have increased 78%, proving costly for both employers and employees (8). In 2007, average annual premiums for employer-sponsored coverage were $4,479 for single coverage and $12,106 for family coverage (8). Indirect costs for employers associated with poor employee health, including absenteeism, presenteeism, disability, or reduced work output, may be several times higher than direct medical costs (9). Productivity losses related to personal and family health problems cost US employers $1,685 per employee per year, or $225.8 billion annually (10).

Health-related programs, policies, and benefits proven to prevent disease and promote health are available to employers. The *Guide to Community Preventive Services* (www.thecommunityguide.org) summarizes many effective health promotion interventions applicable to worksite settings (11). However, studies suggest that many employers are not purchasing or implementing these evidence-based interventions (12). Possible reasons include cost, lack of understanding of health issues and effective interventions, inadequate staffing or capacity to implement programs, and a lack of publicly available tools and resources. Many of these reasons are particularly relevant for small- to medium-sized companies (13). Furthermore, the strategies companies use to address employee health vary by available resources, management and employee needs and interests, and priority health issues.

CDC has a stake in helping employers overcome these barriers and developing specific workplace interventions to assist employers. We present key lessons learned from a collaborative project with Cargill to build Cargill's health promotion capacity and address employer health concerns and barriers through a comprehensive workplace health assessment. Our purpose was to engage Cargill leadership and employees, determine Cargill's priority employee health issues and cost drivers, provide evidence-based recommendations for establishing a workplace health program, and provide technical assistance and guidance to designated staff to build Cargill's internal capacity. CDC worked closely with a Cargill liaison to identify areas where the project could be tailored to the specific needs of the workplace, provide access to multiple data sources to form a comprehensive picture of employee health, and to develop a knowledgeable internal advocate for Cargill's health promotion efforts.

## Context

In the fall of 2006, the CDC Foundation brought CDC and Cargill staff together to collaborate on a workplace health promotion project. CDC partnered with Cargill on a 9-month project aimed at building company health promotion capacity by engaging management and employees, describing priority health issues for which Cargill could take action, and identifying opportunities for and barriers to health promotion at 1 of their local worksites. Cargill, headquartered in Minneapolis, Minnesota, is an international provider of food, agricultural, and risk management products and services. Cargill has 158,000 employees located in 66 countries. RTI International provided analytic expertise, project management, and experience in workplace health programs.

CDC is interested in developing interventions that will allow employers, states, and communities to work together to improve health outcomes for employees and their families. One strategy for developing these interventions is to partner directly with employers to create and test processes, such as conducting environmental audits or reviewing health claims, that result in useful information for planning, implementing, and evaluating workplace health promotion interventions; building employer capacity; and reducing individual and organizational barriers to workplace health promotion.

Cargill was interested in combining their business experience with CDC's expertise in health promotion to develop and test protocols for a comprehensive workplace health assessment in 1 local worksite and to develop knowledge and skills among Cargill staff. Cargill currently has a limited workplace health focus, and health promotion efforts in Cargill worksites are not integrated. The assessment process is a critical first step in establishing a workplace health program. It helps to identify the current status of employee health and to create a plan for initiating or enhancing a workplace health program. The assessment was designed to provide a company roadmap for gauging priority health issues and cost drivers and to recommend specific actions and interventions for establishing a local-level workplace health program.

CDC staff outlined the following 4 project goals that Cargill adopted and supported:

1. Engage diverse groups of Cargill personnel, including leadership, human resources staff, environmental health and safety staff, and employees, as well as community partners able to assist with Cargill's workplace health program.
2. Describe employee health status and the strengths and weaknesses of Cargill's health-related programs, policies, and benefits through a workplace health assessment.
3. Aid in the development of an action plan for improving Cargill employees' health by identifying 3 to 5 priority actions that Cargill could undertake within the next year.
4. Increase Cargill staff capacity through knowledge and skill-building and active participation in all aspects of the project.

## Methods

We first identified a Cargill liaison to work closely with CDC staff throughout the project. Company leaders selected a senior manager from the Cargill corporate human resources department to be the liaison because of this person's background in benefit design and knowledge of personnel issues. The liaison was instrumental in identifying 1) a local intervention site by discussing the project opportunity with potential worksites within the company, 2) appropriate departmental contacts with various levels of worksite responsibility for input, 3) existing health-related data, 4) the need for additional data, 5) communication strategies to gain employee participation, and 6) logistical support for conducting site visits. We developed a solid working relationship with the Cargill liaison through regular conference calls to troubleshoot issues and build internal capacity through knowledge and skill development by explaining the public health rationale and science base behind the assessment approach and protocols. The liaison's role was critical to meeting the project goals of engaging a diverse group of Cargill personnel, conducting the workplace health assessment, and building Cargill's capacity.

CDC staff worked closely with the Cargill liaison to select an appropriate intervention site. They sought employees who were excited to participate, were eager to take action based on CDC recommendations, and understood their efforts would provide models for future Cargill efforts in other worksites throughout the company. A soybean processing site in Sidney, Ohio, was chosen for the following reasons:

- The site leadership was engaged and supported developing a workplace health promotion program.
- The site had experience with health promotion activities but little current activity.
- The site size of 470 employees and demographic characteristics (a combination of sales, administrative staff, engineers, and operators) allowed for a comprehensive assessment and provided a representative sample whose analysis could be applicable to other company sites.
- The site involved 3 separate business units that provided information regarding efforts to integrate data and activities for the same business units at other Cargill sites.
- The site provided an opportunity to assess and recommend actions for a manufacturing site that operates 24 hours per day, 7 days per week, with many employees working 12-hour shifts that rotate between days and nights.

After selecting the intervention site, CDC staff began a 6-week planning phase in January 2007 that included several meetings to introduce the project to key stakeholders from the Cargill corporate office and the local demonstration site's 3 business units to describe the project goals, process, timeline, and expectations. The meetings enabled management to describe the project to employees and elicit interest and participation. The meetings laid the foundation for achieving the project's goals by engaging diverse Cargill personnel and explaining how the workplace health assessment would help develop recommendations for a plan. These meetings resulted in the establishment of regular communications that included home mailings, posts to worksite bulletin boards, and information kiosks to keep managers and employees informed about the project's purpose and scope, progress being made, and outcomes (Appendices A and B).

In March 2007, CDC staff conducted a workplace health assessment during 2- to 3-day site visits to each of the 3 business units to develop an in-depth understanding of the health of Cargill's workforce and workplace. Portions of the assessment built off of CDC's Swift Worksite Assessment and Translational approach (14). The assessment included 6 focus areas:

- Interviews with Cargill management and employees (Appendix C) to:
  1. Learn the company culture and how it might be leveraged or changed for health promotion.
  2. Learn how to build on past successes and experiences and use employee interests and management support for workplace health programs by recommending strategies that would work for Cargill's employees.
  3. Learn about the relationship between the local site and corporate headquarters, recognizing that some recommended strategies could be implemented at the corporate level and consistently adopted for all sites while others were at the local site leadership's discretion and may not be relevant or need to be tailored to the individual worksite.
- An assessment of the physical environment that would support or hinder employee health promotion activities to learn the business operations of what they did and how they did it, recognizing work organization has a relationship to health behavior (Figure).
- A survey of employee health status and risks.
- An analysis of health care and pharmaceutical claims and their costs.
- An analysis of the number and types of work-related injuries.
- An analysis of employee time and attendance.

**Figure.**
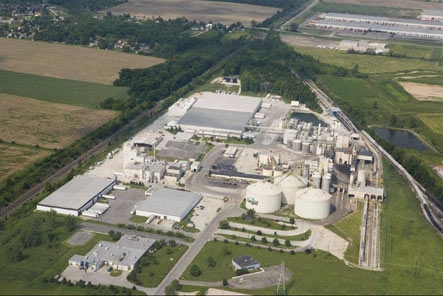
Photo of Cargill Worksite, a Soybean Processing Plant in Sidney, Ohio, CDC-Cargill Workplace Health Promotion Project, 2006-2007.

These analyses informed CDC and Cargill staff about current programs (eg, employee safety), policies, benefits, and environmental supports that could be leveraged. They also identified gaps and points of integration for the business units.

Data not available during the site visits, such as health claims information, was collected and analyzed during the next several months. CDC staff prepared reports describing employee health status, health risks, and cost drivers, as well as a report that provided evidence-based strategies and actions that Cargill could take to improve and promote employee health, form an action plan, and build capacity for the business units.

The project culminated with a 2-day retreat in August 2007 where assessment methods and findings were presented and key next steps and priority actions for the local management team and key employees were outlined (Table). CDC staff stressed implementing interventions that would simultaneously address multiple risk factors and chronic conditions. Our recommendations focused on structuring efforts to improve employee health for the 3 business units combined rather than establishing separate programs, policies, or incentives. We emphasized addressing priority health risks, based on high prevalence or cost, where an effective evidence-based strategy or intervention existed. These recommendations were proposed to ensure the efficient use of limited resources.

During the retreat, CDC staff obtained feedback about Cargill staff's overall impressions of the process, organization, and clarity of the findings and recommendations as well as their utility and practical application, which were well received. Several participants felt the retreat focused too heavily on the methodology, which limited the amount of time to discuss the assessment findings. The project scope did not include selecting and implementing specific health promotion interventions; therefore, we are unable to determine what outcomes have resulted since the assessment concluded. A highlight for Cargill was the invitation of local community health organizations by CDC to participate in the retreat and describe how they could provide additional capacity and assist Cargill's efforts as they planned their program.

During the retreat, CDC staff also discussed with Cargill staff what key health messages, findings, and recommendations to communicate to employees and conducted a visioning exercise, which was popular, to frame the program's development (ie, create a vision and mission for the program, Appendix B). Throughout the project, Cargill learned how the workplace can support and facilitate healthy behaviors for employees and gained knowledge and skills of how to assess, plan, implement, and evaluate a workplace health program. The retreat provided a forum to understand the strengths and weaknesses identified in the assessment and discuss actions necessary to build a successful workplace health program for Cargill's employees.

## Consequences

A key component of this public-private partnership was the relationship between the project leaders of CDC and Cargill. The Cargill liaison played a vital role in orienting CDC staff to the company and workplace, coordinating access to personnel and data for a complete assessment, and providing internal capacity for workplace health promotion efforts. CDC provided technical assistance to many Cargill managers and leaders regarding building workplace health programs using data to guide decision-making and identifying where Cargill could build from their current culture and infrastructure (eg, data systems). CDC educated Cargill about public health principles they were unfamiliar with, such as risk factors, population-based interventions and their evidence base, and community linkages.

The project involved multiple business units in a setting unfamiliar to CDC staff. The Cargill liaison coordinated and scheduled site visits and meetings, making efficient and effective use of the time available. Although a well-defined protocol was established to conduct uniform site visits for each business unit, flexibility was critical because each visit was unique because of differences in business unit operations, job tasks, and work environments. The liaison worked with CDC staff to make necessary adjustments to the protocol. CDC staff did not know the site's employees and leaders, so having the liaison identify the appropriate people to be interviewed was vital in expediting the site visit scheduling. Some business unit functions, such as environmental safety and human resources, were shared across the site. The liaison understood these site-wide functions and streamlined the site visits to avoid duplication. CDC staff learned that, although some functions are shared throughout the site, they are implemented differently within each business unit, which can lead to confusion and perceived disparities among employees. We recommended establishing a uniform program for all business units rather than creating separate programs, policies, or incentives for each to provide equal access and opportunity to health promotion activities.

A review of existing health-related data was also conducted. Cargill, like other businesses, has multiple data information systems from which health-related information can be collected. Some systems are maintained by third parties, and most contain specific nomenclature and variables not immediately intuitive and accessible to external users. The Cargill liaison was able to coordinate and provide timely access to key data, such as working with health-plan, third-party administrators for claims data; human resources for time and attendance data; and environmental safety staff for injury data. The liaison learned from CDC staff how integrating multiple pieces of data from individual data systems during an assessment could provide a more detailed picture of employee health issues. For example, we learned that many of the nutrition choices being made by employees were influenced by the choices available through worksite vending machines, the types of nearby eateries, and job requirements that made it necessary for employees to stay close to their work stations during shifts, limiting where and what types of food could be consumed. CDC staff recommended a healthy foods meetings policy (ie, at a minimum, 1 healthy food option must be made available during a meeting at which food is served) and making available healthier vending foods.

As the project progressed, education became a routine part of the relationship between CDC and Cargill staff, strengthening it and helping achieve the project goal of building internal Cargill health promotion capacity. Through CDC technical assistance, the Cargill liaison gained information and skills related to workplace health promotion and became an in-house expert who could educate others, gain leadership support, and build a culture of health critical to Cargill's long-term program sustainability. The liaison's knowledge, skills, and understanding of the process gained by being a constant throughout the project provided continuity, maintained momentum, and mitigated the effects of staff changes.

CDC staff also recommended designating a workplace health coordinator to design and implement programs recommended from the assessment. Through our technical assistance calls, Cargill staff recognized the value of having a coordinator focused on these efforts and created a position at the Sidney site with access to corporate headquarters. The coordinator would direct the workplace health program, continue to build the local infrastructure and capacity, model successes that may be applied in other company locations, and influence corporate employee health decisions. To further support the workplace health coordinator, CDC staff recommended establishing a wellness committee with representatives from multiple departments and job types because we learned from the assessment that capturing broad employee interests and needs, achieving broad engagement, and improving communications were barriers to overcome in creating a successful program. Cargill has since hired the coordinator and formed the committee. One of this team's first activities is an employee event involving community organizations introduced to Cargill during the retreat to provide information and education on healthy lifestyles and conduct health risk assessments to inform employees of and recommend actions for employees' individual health risks.

## Interpretation

Businesses are complex organizations where it often takes employees months or years to learn the company culture, customs, policies, and practices. As external assessors, CDC staff had little time to become knowledgeable about Cargill's employees and how the business operated and functioned. The ability of CDC staff to conduct a useful and relevant workplace health assessment was contingent on having an individual within the company to facilitate the process. Our relationship with the company liaison developed and strengthened throughout the 9-month project and resulted in a level of trust through regular, open, and honest communication that allowed our project to meet its goals. The trust established allowed access to and participation from key stakeholders, access to data and facilities for the assessment, the free exchange of information and ideas to develop practical recommendations for an action plan, and troubleshooting issues. The relationship was pivotal to building momentum, interest, and commitment to fully understanding employee and employer needs within the context of the corporate culture, generating credibility and buy-in to the assessment process, and positioning the liaison to be a better advocate for workplace health within the company.

The openness provided by Cargill managers and employees was critical to the richness of the findings and practicality of the recommendations. It resulted from a good working relationship between CDC and Cargill staff who were focused on common goals of improving employee health. The relationship developed by engaging many managers and employees, creating excitement for the opportunity to build a workplace health program, and devoting substantial time to it.

Identifying a company liaison to work closely with an external assessment team is critical to conducting a successful workplace health assessment and building company health promotion capacity. Employers undertaking workplace health assessments should consider a company liaison at a senior level to command the attention of leadership, have the necessary influence to keep the project moving forward, and address issues with managers and employees at the worksite. A senior-level person would be able to see lessons learned from a local site and determine what may be applicable to other company sites, whereas someone focused on the site level without a companywide viewpoint may not have this perspective. They also will be able to develop a companywide strategy based on local demonstrations and enact that change, where local leaders may not have that influence. They can create balance between a corporate strategy and local level adaptation and innovation of health promotion activities.

## Acknowledgments

This project was funded through a partnership between CDC, the CDC Foundation, and Cargill. We acknowledge the contributions of Jennifer Alexander, Olga Khavjou, and Pam Williams-Piehota of RTI International and Jenelda Thornton of CDC in data collection and analysis. We thank all of the Cargill managers and employees at both the local site and corporate headquarters for their interest and generosity in sharing their experiences and time throughout the worksite assessment process.

## Figures and Tables

**Table.** CDC-Cargill Workplace Health Retreat Agenda, Sidney, Ohio, 2006-2007

Day 1: Thursday, August 30		
**Time**	**Topic/Activity**	**Goals/Details**
9:00 AM	Day 1 Kick-off	Welcome and Introductions; Days 1 and 2 Agenda Review; Establish meeting ground rules
9:30 AM	Recap of Assessment Activities	Recap of Project Objectives/Expectations; Summary of Project Activities to Date
10:00 AM	Assessment Findings	Presentation of Qualitative and Quantitative Findings for Site; Review of Methodology as Appropriate
11:00 AM	Questions and Answers/Energy Break	
11:30 AM	LunchSidney Working Group Discussion	Reflection on Findings (Format: Small Group Discussions/Large Group Report Out)
12:30 PM	Recommendations	Presentation of Recommendations
1:30 PM	Questions and Answers/Energy Break	
2:00 PM	Sidney Working Group Discussion	Reflections on Recommendations (Format: Small Group Discussions/Large Group Report Out)
3:30 PM	Day 1 Wrap-up	Identify/Capture Day 1 Actions and Outcomes; Capture Open Questions; Set Expectations for Day 2 Working Group
4:00 PM	Adjourn	

Day 2: Friday, August 31		
**Time**	**Topic/Activity**	**Goals/Details**
7:30 AM	Day 2 Kick-off	Review Day 1 Actions and Outcomes; Address any Open Questions From Day 1; Day 2 Agenda Review
7:45 AM	Setting Partnership Expectations	Outline Considerations for Maximizing Community Partnerships
8:00 AM	Sidney Working Group Discussion	Begin Action Planning — Identify Next Steps; Articulate Program Vision/Mission; Priority Setting Activity
8:00 AM	Community Partner Plant Tour	Tour of Sidney Site for Community Partner Representatives
10:00 AM	Community Partner Roundtable Kick-off	Introductions; Cargill Overview
10:30 AM	Community Partner Roundtable	Community Partner Presentations (not to exceed 15 minutes)
11:30 AM	Lunch	
12:15 PM	Day 2 Wrap-up	Capture Meeting Actions and Outcomes; Capture Open Questions/Deliverables
1:00 PM	Adjourn	

Abbreviation: CDC, Centers for Disease Control and Prevention.

## Appendices

### Appendix A – Employee Communications CDC-Cargill Workplace Health Project Announcement

February 2007

We are excited to announce that Cargill will be kicking off a new project aimed at making our company a healthier place to work — and helping you enjoy all the benefits of good health.

**Why is This Important?**

A healthy workforce benefits everyone: you; your family, friends, and co-workers who care about you; and the company's bottom line.

At Cargill we've worked hard to protect the safety of our workforce. We'd like to build on that success and provide the support you need to also protect **your** **health**. Why? Because a healthy and safe workforce is critical to Cargill's ability to deepen relationships with customers and fulfill our promises to the communities we serve. We think *Working on Wellness* is a great place to start.

**What is *Working on Wellness*?**

*Working on Wellness* is a partnership between Cargill and its employees to improve employee health and quality of life through preventive education and health risk management services. Desired results will positively impact employee engagement and productivity, which encouraging appropriate healthcare utilization.

**Who is Involved in *Working on Wellness*?**

We are delighted to be partnering with the US Centers for Disease Control and Prevention (CDC), the nation's prevention agency, in our wellness project. With CDC's assistance, we will be performing a *Working on Wellness* assessment at Sidney. The work will involve all three Business Units at our location and take place over the next 9 months.

The goals of the assessment are:

Structure wellness efforts to improve employee health Identify strengths and weakness of our policies and programs that impact employee health Recommend activities for employees to maintain and improve their health Increase Cargill staff's capacity in the area of wellness promotion

**What Can You Expect?**

Over the next few months a team from the CDC will be in Sidney facilitating the assessment. We hope to learn much during this time, and your participation is key to our learning process. The worksite assessment will involve:

*Interviews with Managers and other Employees*
Interviews will be a combination of one-on-one and small-group discussions. The goals of the interviews are to gain a better understanding of your impressions of success, and to identify opportunities that will lead to a successful wellness program in our company. A trained interviewer from the CDC will conduct the interviews. We fully encourage your participation in the interviews if you are asked, and we assure that the team will respect your privacy and confidentiality.
*An Environmental Assessment*
The assessment will consider our buildings, including stairwells, work areas, and locker rooms, as well as common areas, like our break rooms. It will take into account how we use our signs and bulletin boards for communicating around our worksites. The assessment will also include our grounds and the communities where we live.
*Direct Interaction and Input*
The team will talk with you in your work environment. Building a culture of health requires your open and honest input. We need to hear from you!

**How Long Until We Will See Action Being Taken?**

We expect that the assessment will continue into the fall of this year. You can expect to hear updates as the project moves ahead.

**If You Have Questions**

Contact [Name] in Human Resources — either directly or through your supervisor — if you have questions or if you would like to volunteer to help with the project.

### Appendix B – CDC-Cargill Workplace Health Project Employee Communications Follow-Up Brochure

The brochure used in this study is available for download as a Microsoft Word document. You must have Microsoft Word to use this file.

**Download the Communications Follow-Up Brochure** (DOC 43k)

### Appendix C – Sample Business Unit Site Visit Schedule

The schedule used in this study is available for download as a Microsoft Word document. You must have Microsoft Word to use this file.

**Download the Site Visit Schedule** (DOC 111k)
