# Supplementary material for: Building Company Health Promotion Capacity: A Unique Collaboration Between Cargill and the Centers for Disease Control and Prevention
Source: Prev Chronic Dis. 2009 Mar 15;6(2):A62. (PMC2687868)
Supplement: Supplementary file 1 [file 08_0198_01.doc]

## **thank you, sidney employees!**

In January we kicked off a project, Working on Wellness (WOW), aimed at making Cargill in Sidney a healthier place to work, helping you and your family enjoy all the benefits health, and helping the company ability to serve its customers and the local community.

Throughout the month of March, a team from the Centers for Disease Control and Prevention (CDC), the nation’s prevention agency, were in Sidney talking to managers and employees about our impressions of what a successful wellness program at our Company would look like. We thank you for your willingness to share your thoughts with this team, your openness, and for your time showing them around our location so that the CDC team could also look at the physical work environment and how future programs may integrate with what we already do on a daily basis. Through these site visits, the CDC now has a much better understanding of who we are, what we do, and what are health issues are.

We also appreciate the time many of you took to complete the anonymous survey about your current health status and health-related behaviors. Great job! This information will help the assessment team tailor their recommendations to fit our health issues and concerns here in Sidney.

If you didn’t have the opportunity to talk to a member of the CDC team, or you have other ideas you would like to share, it’s not too late. If you have an interest in being actively involved in the activities related to the development and promotion of our program in Sidney, by all means, let us know! Look inside for information on how you can continue to be involved.

We again thank you for all your input and involvement in this process thus far and we look forward to being able to build a health promotion programs together with you later this year, offering you options for living a healthier lifestyle.

*Sincerely,*

*The Sidney Leadership Team*

**Thank you**

for helping to create a program to support making Cargill a healthier place to work!

Contact [Name] in Human Resources – either directly or through your supervisor – if you have questions, if you would like to provide input to the assessment team, or if you are interested in being involved in the wellness planning committee related to the development and promotion of the Working on Wellness Program.

# Cargill working on wellness

## what is working on wellness?

Working on Wellness is a partnership between Cargill and its employees to improve employee health and quality of life through preventive education and health risk management services. Desired results will positively impact employee engagement and productivity, while encouraging appropriate healthcare utilization.

## why is this important?

A healthy workforce benefits everyone: you; your family, friends, and co-workers who care about you; and the company’s bottom line.

At Cargill we’ve worked hard to protect the safety of our workforce. We’d like to build on that success and provide the support you need to also protect **your** **health**. Why? Because a healthy and safe workforce is critical to Cargill’s ability to deepen relationships with customers and fulfill our promises to the communities we serve. We think “Working on Wellness” is a great place to start.

## what is a wellness program?

Wellness programs are organized employer sponsored activities located on or off-site intended to assist employees and their families in making voluntary behavior changes which reduce their health and injury risks, improve their health consumer skills, and enhance their individual productivity, physical, and emotional well-being...

As part of our Wellness Program, Cargill is considering:

- Programs such as nutrition, physical activity, stress management, or smoking cessation programs
- The Environment, like our physical work areas, the Sidney worksite campus, and our local community which creates and supports a culture of health
- Policies, such as tobacco use, and what is offered in our vending machines.
- Benefits and Incentives for participation or meeting health goals to make the healthy choice the easy choice

## how long until we see action being taken?

Much work has already been done. Along with the information we have gathered from the survey and talking to you, we have been hard at work with the CDC looking at other data sources like our health claim experience, absenteeism rates, and safety records. All this information will assist CDC in tailoring their recommendations to our worksite. We expect that the CDC’s recommendations will be presented this summer and implementation of the program will begin to take shape in the fall.

## Who is involved in working on wellness?

We are delighted to be partnering with the U.S. Centers for Disease Control and Prevention (CDC), the nation’s prevention agency, in our wellness project. The work involves all three Business Units at our location. You can be involved too – If you participated in the survey about your health status and behaviors, or you spent time with the CDC’s assessment team in March, you already have!

We are looking for interested employees from each business unit to participate in a wellness planning committee to review and take action on the CDC recommendations to us. There is also the opportunity to continue to provide feedback and input on our effort by filling out a comment card and placing them in the blue WOW boxes that we used for the employee survey. The planning committee will routinely collect and review those comments.

It’s not too late to provide more input or to get involved in the activities related to the development and promotion of our program in Sidney.
