# Supplementary material for: Building Company Health Promotion Capacity: A Unique Collaboration Between Cargill and the Centers for Disease Control and Prevention
Source: Prev Chronic Dis. 2009 Mar 15;6(2):A62. (PMC2687868)
Supplement: Supplementary file 2 [file 08_0198_02.doc]

| **APPENDIX C: Sample Business Unit Site Visit Schedule** | | |  |  |  |
| --- | --- | --- | --- | --- | --- |
| **Cargill-Sidney Site Visit Assessment** | | |  |  |  |
| *Agenda* |  |  |  |  |  |
| BU Focus: | **Business Unit #2** | |  |  |  |
| Dates: | **March 7- March 9** | |  |  |  |
|  |  |  |  |  |  |
| **Wednesday, March 7, 2007** | | |  |  |  |
|  |  |  |  |  |  |
| **Time** | **Est. Time** | **Item** | **Cargill Contributors/Attendees** | | **Location** |
| 8:00 AM | 1-2 hrs | Participate in Professional Development Training Program | Business Manager | | Holiday Inn |
| 10:30 AM | 30 min | Orientation Meeting | General Manager | | Small Conf Room |
|  |  |  | Department Superintendent | |  |
|  |  |  | Site Superintendent | |  |
|  |  |  | Human Resources Manager | |  |
|  |  |  | Environmental Health and Safety (EHS) Coordinator | |  |
| 11:00 AM | 1 hr | Leadership Interview | General Manager | |  |
| 12:00 PM | 1 hr | Lunch |  |  |  |
| 1:00 PM | 1 hr | Leadership Interview | Site Superintendent | | Small Conf Room |
|  |  |  | Maintenance Leader | |  |
| 2:00 PM | 1 hr | Leadership Interview | Department Superintendent | | Small Conf Room |
|  |  |  |  |  |  |
| 3:00 PM | 30 min | BREAK |  |  |  |
| 3:30 PM | 1 hr | General Interview | Logistics Supervisor | | Small Conf Room |
|  |  |  | Accounting Supervisor | |  |
|  |  |  | Accounting Supervisor | |  |
| 5:00 PM |  | 12 hour Shift Change - Employee Observation, Informal Interviews |  | |  |
|  |  |  |  |  |  |
| **Thursday, March 8, 2007** | | |  |  |  |
|  |  |  |  |  |  |
| **Time** | **Est. Time** | **Item** | **Cargill Contributors/Attendees** | | **Location** |
| 8:30 AM | 10-15 min | Check-in Meeting | General Manager | | Small Conf Room |
|  |  |  | Department Superintendent | |  |
|  |  |  | Site Superintendent | |  |
|  |  |  | Human Resources Manager | |  |
|  |  |  | EHS Coordinator | |  |
| 9:00 AM | 30 min | Hold for Production Meeting - Production/Supervisors not available | |  |  |
| 9:30 AM | 1 hr | General Interview | Production Supervisor | | Small Conf Room |
|  |  |  | Production Supervisor | |  |
| 10:30 AM | 30 min | BREAK |  |  |  |
| 11:00 AM | 1 hr | Engagement Team Interview | Logistics Specialist | | Small Conf Room |
|  |  |  | Plant Maintenance | |  |
|  |  |  | Operator | |  |
| 12:00 PM | 30 min | BREAK |  |  |  |
| 12:30 PM | 1 hr | Safety Team Interview | EHS Coordinator (Team member) | | Small Conf Room |
|  |  |  | Production Supervisor (Team member) | |  |
|  |  |  | Department Superintendent (Team member) | |  |
|  |  |  | Production Operators on Shift (Team members) | |  |
| 1:30 PM | 2-3 hrs | Environmental Assessment | EHS Coordinator -Sidney Site | |  |
| 4:00 PM | 1 hr | EHS Interview | EHS Coordinator -Sidney Site | |  |
| 5:00 PM |  | Shift Change/Dinner BREAK |  |  |  |
| 12:00 AM | 1 hr | Night Shift, Observation of Employees, Informal Interviews | Site Crew leader on shift | |  |
|  | | | | | |
| **Friday, March 9, 2007** | |  |  |  |  |
|  |  |  |  |  |  |
| **Time** | **Est. Time** | **Item** | **Cargill Contributors/Attendees** | | **Location** |
| 8:30 AM | 10-15 min | Check-in Meeting | General Manager | | Small Conf Room |
|  |  |  | Department Superintendent | |  |
|  |  |  | Site Superintendent | |  |
|  |  |  | Human Resources Manager | |  |
|  |  |  | EHS Coordinator | |  |
| 8:45 AM | 1 hr | HR Interview | Human Resources Manager | |  |
|  |  |  |  |  |  |
| 10:00 AM | 1 hr | Recognition Team (Office staff) | Accounting Associate | | Small Conf Room |
|  |  | Interview | Merchandising Assistant | |  |
|  |  |  | Merchant | |  |
|  |  |  | Sales Coordinator | |  |
|  |  |  | Accounting Supervisor | |  |
|  |  |  |  |  |  |
| 11:00 AM | 30 min | BREAK |  |  |  |
| 11:30 AM | 1 hr | Recognition Team (Production staff) | Crew leader | | Small Conf Room |
|  |  | Interview | Operator | |  |
|  |  |  |  |  |  |
| 12:30 PM |  | Hold open for HR/EHS follow-up |  |  |  |
